# Health and Athletics

**Published:** 1898-02

**Authors:** 


### SELECTIONS.

### Health and Athletics.

What is precisely meant by the words "athletic" and "healthy?" asks the *St. James Gazette*. The former may be dismissed in a word or two. By an "athlete" is meant the "trained" man, he who undergoes special preparation for a certain object. Of course, an untrained man may be an athlete, but the word is generally used in the more special sense. Therefore, under this category will not be included those who "go in for exercise" as distinguished from "athletes." And now what is it to be "healthy?" It is easier to say what it is not than what it is. A man may be a fleet runner, a fine oar, splendidly muscular, and agile, yet need not be "healthy" as the writer understands the word. It sounds like a paradox, but a man may be able to lift tremendous weights, to put on a terrific spurt at the end of a long and punishing race, to "send in his left" with amazing force and rapidity, and yet be absolutely delicate. In short, physical strength and health, often as they have been considered as synonymous, have very little to do with one another.

What then do we mean by being "healthy?" Briefly, it may be said that the healthy system is that of which all the parts work in perfect harmony. Such a system requires a very great shock, indeed, to throw it altogether out of gear. It has great recuperative powers, which tend to make up any deficiency caused by carelessness or disease. The healthy system possesses, moreover, great elasticity, and works vigorously to throw off any evil germs before they have got a secure footing. Health is a good deal more than the absence of disease; it is emphatically an active condition. And its distinguishing sign is adaptability. The really healthy man is he who, after catching a slight cold, goes to bed, sleeps soundly, and wakes up perfectly well in the morning; who easily recovers from any unusual strain upon his organization; who can stand extremes of climate, privation and hardship; who is not permanently upset by even a violent departure from his ordinary course of life; who does not trouble much about what he eats and drinks, so long as it is wholesome; this is a healthy man. He may or may not be what is rather erroneously termed "strong;" that is a trifling matter. Whether he is strong in another and truer sense of the word may be judged by those who know how often your great, strong, athletic fellow succumbs to a complaint which the so-called "weakling" manages to struggle through.

To return to the athlete. Can any one who has any real knowledge of the subject deny that, judged by the standard which has been laid down, the athlete will, as a rule, be found wanting? Let us take the oarsman as typical of the amateur athlete. To look at the young 'varsity oar is to get an impression of a man thoroughly hardened to wind and weather, to whom such things as colds and chills were unknown. But is it so? Most emphatically, no! How often do we hear of his being temporarily incapacitated through some trifling mishap or indisposition? How often is he laid up? How often "off color?" How often down with something serious? And especially is this noticeable when he is a little older and has left off training regularly. If one casts one's memory back through the years, how many once brilliant athletes can we call to mind as having broken down and died untimely deaths?

We can think of many, but the names of those who have not died, but have just managed to keep alive after struggling through long and painful illness, is legion. Allusion is not here made to specific complaints—such as heart disease—brought on by over-exertion, although this is not infrequent. But what is meant is rather the general weakening of the system, the loss of that "spring" and energy which are so essential to a man who has to fight against illness. As with the oarsman, so with the running man. The latter is particularly prone to little injuries to his feet and legs, and in him the results of training are shown very graphically. For an ordinary man the cure of a sprained ankle or strained tendon is a matter of weeks; for the trained man it often means being for months on the shelf. So long as things shape a normal course he appears to be in perfect health; directly the unexpected occurs one sees that he does not possess the stamina and recuperative energy he so badly needs.

